# Comparison of lung disorders following intratracheal instillation of polystyrene microplastics with different surface functional groups

**DOI:** 10.1093/joccuh/uiaf006

**Published:** 2025-02-03

**Authors:** Taisuke Tomonaga, Hidenori Higashi, Hiroto Izumi, Chinatsu Nishida, Kazuma Sato, Yuiko Nakamura, Toshiki Morimoto, Yasuyuki Higashi, Takuma Kojima, Kazuo Sakurai, Kazuhiro Yatera, Yasuo Morimoto

**Affiliations:** Department of Occupational Pneumology, Institute of Industrial Ecological Sciences, University of Occupational and Environmental Health Japan, 1-1 Iseigaoka, Yahata-nishi-ku, Kitakyushu, Fukuoka 807-8555, Japan; Department of Environmental Health Engineering, Institute of Industrial Ecological Sciences, University of Occupational and Environmental Health Japan, 1-1 Iseigaoka, Yahata-nishi-ku, Kitakyushu, Fukuoka 807-8555, Japan; Department of Occupational Pneumology, Institute of Industrial Ecological Sciences, University of Occupational and Environmental Health Japan, 1-1 Iseigaoka, Yahata-nishi-ku, Kitakyushu, Fukuoka 807-8555, Japan; Department of Environmental Health Engineering, Institute of Industrial Ecological Sciences, University of Occupational and Environmental Health Japan, 1-1 Iseigaoka, Yahata-nishi-ku, Kitakyushu, Fukuoka 807-8555, Japan; Department of Occupational Pneumology, Institute of Industrial Ecological Sciences, University of Occupational and Environmental Health Japan, 1-1 Iseigaoka, Yahata-nishi-ku, Kitakyushu, Fukuoka 807-8555, Japan; Department of Occupational Pneumology, Institute of Industrial Ecological Sciences, University of Occupational and Environmental Health Japan, 1-1 Iseigaoka, Yahata-nishi-ku, Kitakyushu, Fukuoka 807-8555, Japan; Department of Respiratory Medicine, University of Occupational and Environmental Health Japan, 1-1 Iseigaoka, Yahata-nishi-ku, Kitakyushu, Fukuoka 807-8555, Japan; Department of Respiratory Medicine, University of Occupational and Environmental Health Japan, 1-1 Iseigaoka, Yahata-nishi-ku, Kitakyushu, Fukuoka 807-8555, Japan; Department of Chemistry and Biochemistry, The University of Kitakyushu, 1-1 Hibikino, Wakamatsu-ku, Kitakyushu, Fukuoka 808-0135, Japan; Department of Chemistry and Biochemistry, The University of Kitakyushu, 1-1 Hibikino, Wakamatsu-ku, Kitakyushu, Fukuoka 808-0135, Japan; Department of Respiratory Medicine, University of Occupational and Environmental Health Japan, 1-1 Iseigaoka, Yahata-nishi-ku, Kitakyushu, Fukuoka 807-8555, Japan; Department of Occupational Pneumology, Institute of Industrial Ecological Sciences, University of Occupational and Environmental Health Japan, 1-1 Iseigaoka, Yahata-nishi-ku, Kitakyushu, Fukuoka 807-8555, Japan

**Keywords:** lung disorders, surface functional groups, polystyrene, microplastic, intratracheal instillation, rat

## Abstract

**Objectives:**

Microplastics have been detected in the atmosphere, raising concerns about their impact on the lungs. There have been reports on the effects of surface functional groups in evaluating the physicochemical properties of microplastics, but no reports have evaluated their chronic effects. We performed intratracheal instillation in rats to evaluate the acute and chronic effects on the lungs of microplastics with different surface functional groups.

**Methods:**

Unmodified, NH_2_-modified, and COOH-modified polystyrene particles with a particle size of 1 μm were intratracheally instilled into the lungs of rats. Rats were dissected at 3 days, 1 week, 1 month, 3 months, and 6 months after exposure to analyze inflammatory cells and lung injury factors in bronchoalveolar lavage fluid (BALF) and to observe histopathological findings in the lungs.

**Results:**

A significant increase in the number of inflammatory cells in BALF was observed up to 1 week after exposure to the NH_2_-based modified polystyrene compared with the negative control group. A significant increase was observed 3 days after exposure, and histopathological findings in the lungs also showed an influx of inflammatory cells into the alveolar space in the acute phase, but not in the chronic phase. In in vitro studies using RAW cell lines, NH_2_-based modified polystyrene also induced the highest oxidative stress compared with unmodified and COOH-based modified polystyrene.

**Conclusions:**

These results suggest that these polystyrenes do not have high pulmonary toxicity, although there are differences in toxicity due to differences in surface functional groups only in the acute phase.

## Teaser

Key points

**What is already known on this topic:** There have been reports of acute lung inflammation caused by polystyrene with different functional groups, but no reports on their chronic effects.

**What this study adds:** Amino-modified polystyrene had relatively high acute effects compared with unmodified and carboxy-modified polystyrene, but no chronic pulmonary effects were observed with either polystyrene.

**How this study might affect research, practice, or policy:** The results of this study suggest that lung disorders in the chronic phase caused by differences in the surface functional groups of polystyrene may not be severe.

## Introduction

Environmental pollution by microplastics, mainly in the ocean, has been a concern in recent years, but microplastics have also been confirmed in the atmosphere.^1^^,^^2^ Furthermore, since microplastics have been detected in the lungs of humans and migratory birds,^2^^,^^3^ there are concerns about the biological effects of respiratory exposure. In addition, in the plastic recycling and manufacturing process, high-resolution computed tomography has shown peripheral bronchial thickening, increased interstitial shadows, and elevated serum inflammatory cytokines in workers who handle microplastics,^4^ suggesting that occupational exposure may lead to the development of respiratory diseases. The European Plastics Strategy includes a strategy to reduce plastic waste as well as a strategy to promote plastic recycling,^5^ and it is expected that plastic recycling will progress in the future, increasing the risk of occupational exposure for recycling workers. It is an urgent task, therefore, to evaluate the effects of airway exposure to microplastics not only in the general atmosphere but also in occupational environments.

It is generally thought that inhaled chemicals taken into the lungs are deposited in the lungs, and persistent lung inflammation leads to irreversible fibrosis and tumor formation. Among inorganic chemicals, crystalline silica and asbestos, which have high pulmonary toxicity, are known to deposit in the lungs, causing persistent pulmonary inflammation, fibrosis, and tumor formation.^6^^-^^8^ Considering that microplastics, like inorganic chemicals, do not decompose easily, they may cause pulmonary toxicities if deposited in the lungs for a long time.

Microplastics of various types, sizes, and shapes are observed in the atmosphere, and their physicochemical properties are considered to be involved in various biological effects.^9^^,^^10^ The biological effects of the surface functional groups of microplastics have been reported in cultured cell tests and intratracheal instillation in mice using polystyrene with different surface functional groups, and it has been reported that different surface functional groups produce differences in acute effects.^9^^,^^11^^,^^12^ Generally, a large amount of exposure is required to cause acute effects, and it is unlikely that acute effects will occur given the actual environmental concentrations of microplastics. It is necessary, therefore, to evaluate the effect microplastics have on the lungs when they are deposited in the lungs for a long time; that is, the chronic effects of the surface functional groups of microplastics. There are few reports, however, of the chronic effects of the surface functional groups of microplastics.

We evaluated the chronic effects of differences in the surface functional groups of microplastics by conducting intratracheal instillation in rats using polystyrene with different surface functional groups, and observed them until the chronic stage to evaluate their effects on lung disorders.

## Methods

### Sample preparation

A dispersion of pristine polystyrene (PS-Plain), amino-functionalized polystyrene (PS-NH_2_), and carboxy-functionalized polystyrene (PS-COOH) microplastics with an average size of 1 μm were purchased (PS04001, PA04001, PC04001; Bangs Laboratories, Inc., IN, USA). The purchased polystyrene dispersions of PS-Plain and PS-COOH included 0.1% sodium dodecyl sulfate (SDS), and the PS-NH_2_ included 0.1% Tween 20. In order to maintain a good dispersion condition, each dispersant was used without removing them in this study. Polystyrene dispersion is white, with a specific gravity of 1.06. Approximately 100 mg/mL polystyrene dispersion was suspended in distilled water to a concentration of 2.5 mg/mL for intratracheal instillation in rats. Negative control samples were prepared by removing the polystyrene from each polystyrene dispersion and using the same amount of dispersant as contained in the PS instillation solution. Solvents for the negative control groups of each polystyrene were obtained with each polystyrene dispersion by size filtration using a 220-nm pore filter (PES013022; Membrane Solutions Ltd, TX, USA) or by ultrafiltration (VIVASPIN 6; Sartorius Japan K.K., Tokyo, Japan) with centrifugation at 400 *g* and 4°C for 1 hour. The size distribution of each polystyrene particle in the dispersion was measured by dynamic light scattering (DLS) (ZEN1600; Malvern Panalytical, Ltd, Malvern, UK), and a dried sample of the dispersion was observed by scanning electron microscopy (SEM) by HITACHI S-4500 (Hitachi, Ltd, Tokyo, Japan).

To analyze the physicochemical properties and perform cell culture, we prepared the polystyrene particles extracted from the prepared polystyrene dispersion by alcohol dehydration using ethanol and drying. The polystyrene dispersions were mixed in equal amounts with approximately 25% ethanol and centrifuged at 22518 *g* at 4°C for 5 minutes. The supernatant was removed, increasing concentrations of ethanol were added, and the centrifugation was repeated (approximately 50%, 75%, and 100% ethanol) (09666-85; Nacalai Tesque, Inc., Kyoto, Japan). Each obtained polystyrene powder was suspended in 0.1% Tween 80 water solution.

### Physicochemical characterization of polystyrene dispersions

The zeta potential was measured using a Zetasizer Nano-ZS (Malvern Instruments, Malvern Panalytical Ltd, Malvern, UK) in polystyrene suspended in 0.1% Tween 80 water solution. The pH was also measured using a pH meter (HORIBA, Ltd, Kyoto, Japan) in polystyrene suspended in 0.1% Tween 80 water solution. Fourier transform infrared (FT-IR) spectra were measured on a JASCO FT-IR 4200 spectrometer (JASCO Engineering Co., Ltd, Tokyo, Japan) to investigate the surface properties using the KBr plate method (TabletMaster; JASCO Engineering Co., Ltd, Tokyo, Japan). The resolution was 4 cm^−1^ except where indicated. In the method, only KBr plate was attached to the spectrometer for background measurement, then each polystyrene was placed into the KBr plate and measured for IR spectrum. The background obtained was subtracted from the spectrum of the polystyrene sample as follows: (Spectrum of sample)/(Background spectrum) × 100 = Transmission spectrum. The main transmittance curves of the 3 different particles of PS-Plain, PS-COOH, and PS-NH_2_ corresponded with the typical spectra of polystyrene.

### Animals

Male Fischer 344 rats (8-10 weeks old) were purchased from Japan SLC, Inc. (Shizuoka, Japan). The animals were kept in the Laboratory Animal Research Center of the University of Occupational and Environmental Health for 2-4 weeks with access to free-feeding of commercial diet and water. All procedures and animal handling were done according to the guidelines described in the Japanese Guide for the Care and Use of Laboratory Animals as approved by the Animal Care and Use Committee, University of Occupational and Environmental Health, Japan (animal studies ethics clearance proposal number; AE20-015).

### Intratracheal instillation

We administered 0.4 mL of prepared polystyrene dispersion—doses of 0.2 mg (0.8 mg/kg bodyweight [BW] and 1.0 mg (4.0 mg/kg BW)—to the lungs of rats (12 weeks old) in single intratracheal instillations. The rats were intratracheally instilled under anesthesia by sevoflurane (VIATRIS Japan, Tokyo, Japan) inhalation. Briefly, a laryngeal extension was performed using a laryngoscope blade (MAC1; Rudolf Riester GmbH, Jungingen, Germany), an animal feeding needle (KN-348; Natsume Seisa-kusho Co., Ltd, Tokyo, Japan) was inserted directly into the trachea, and the suspension was manually injected. Then 3 mL of air was inserted into the trachea twice with a syringe via the animal feeding needle. The rats were then allowed to awaken spontaneously and were observed periodically. Each control group received a prepared dispersion with the polystyrene particles removed. Rats were dissected at 3 days, 1 week, 1 month, 3 months, and 6 months after the instillation under anesthesia by isoflurane (VIATRIS Japan, Tokyo, Japan) inhalation.

### Animals following inhalation and intratracheal instillation

There were 5 rats in each exposure and control group at each time point. Body and lung weights were measured under anesthesia by isoflurane inhalation, and then at autopsy blood was removed from the abdominal aorta and the lungs were perfused with normal saline. The right lungs were repeatedly inflated with normal saline under a pressure of 20 cmH_2_O, following fluid recovery 2 times, while the left main bronchus was clamped. Between 7 and 14 mL of the recovered bronchoalveolar lavage fluid (BALF) was collected in collection tubes by free fall, and then the right and left lungs were divided. The homogenized third lobes of the right lungs were used for Heme Oxygenase-1 (HO-1) western blotting after recovery of BALF. The left lungs were inflated and fixed by 10% formaldehyde under a pressure of 25 cmH_2_O for use in histopathological evaluation.

### Cytospin analysis of inflammatory cells and measurement of inflammation-related markers in BALF

BALF was centrifuged at 400 *g* at 4°C for 15 minutes, and the supernatant was transferred to a new tube for measurement of total protein, lactate dehydrogenase (LDH), and cytokines. The pellets were washed by suspension with polymorphonuclear leukocyte (PMN) buffer (137.9 mM NaCl, 2.7 mM KCl, 8.2 mM Na_2_HPO_4_, 1.5 mM KH_2_PO_4_, and 5.6 mM glucose) and centrifuged at 400 *g* at 4°C for 15 minutes. After removal of the supernatant, the pellets were resuspended in 1 mL of PMN (poly-morphonuclear leukocyte) buffer. The number of cells in the BALF was counted by ADAM-MC (AR BROWN Co., Ltd, Tokyo, Japan), after which the cells were splashed on a glass slide using cytospin and fixed and stained with Diff-Quik (Sysmex Co., Kobe, Hyogo, Japan). The numbers of neutrophils and alveolar macrophages were then counted by microscopic observation. The released LDH activity in the BALF supernatant was measured by a Cytotoxicity Detection KitPLUS (LDH) (Roche Diagnostics GmbH, Mannheim, Nordrhein-Westfalen, Germany) according to the manufacturer’s instructions. LDH activity was estimated using a standard curve obtained from known concentrations of recombinant LDH from rabbit muscle (Oriental Yeast Co., Ltd, Tokyo, Japan). Concentrations of cytokine-induced neutrophil chemoattractant (CINC)-1 and CINC-2 in the BALF were measured by ELISA kits (#RCN100, #RCN200, respectively; R&D Systems, Minneapolis, MN, USA). All measurements were performed according to the manufacturer’s instructions.

### Total RNA extraction

The third lobes of the right lungs (*n* = 5 per group per time point) were homogenized while using a QIAzol lysis reagent with a TissueRupotor (Qiagen, Hilden, Germany). Total RNA from the homogenates was extracted using an miRNeasy Mini Kit (Qiagen, Hilden, Germany) following the manufacturer’s instructions. RNA purity and integrity were evaluated by an ND-1000 Spectrophotometer (NanoDrop, Wilmington, NC, USA), and an Agilent 2100 Bioanalyzer (Agilent Technologies, Palo Alto, CA, USA).

### Validation of gene expression data using quantitative real-time polymerase chain reaction

Quantitative real-time polymerase chain reaction (qRT-PCR) was performed as described previously.^13^ Briefly, the total RNA extracted from the lungs at each observation point in each group was transcribed into complementary DNA (cDNA) (High-Capacity cDNA Reverse Transcription Kit; Thermo Fisher Scientific Inc., MA, USA). qRT-PCR assays were performed while using TaqMan (TaqMan Gene Ex-pression Assays; Thermo Fisher Scientific Inc., Waltham, MA, USA) according to the manufacturer’s protocol. Gene expression data were analyzed by the comparative cycle time (ΔΔCT) method. The Assays-on-Demand TaqMan probes and primer pairs were Heme Oxygenase 1 gene (*Hmox1*) (Assay ID Rn00561387_m1), Glutamate-Cysteine Ligase Modifier Subunit gene (*Gclm*) (Assay ID Rn00568900_m1), and Sulfiredoxin 1 gene (*Srxn1*) (Assay ID Rn04337926_g1). All experiments were performed in StepOnePlus Real-Time PCR Systems (Thermo Fisher Scientific Inc., MA, USA). All expression data were normalized to endogenous control β-actin expression (Assay ID Rn00667869_m1) and calculated relative to their gene expression in each negative control.

### SDS-PAGE and western blotting

The third lobes of the right lungs were homogenized with a T-PER tissue protein extraction reagent (Thermo Scientific Inc., Rockford, IL, USA), including protein inhibitor cocktails (P8340; Sigma-Aldrich, St Louis, MO, USA) and complete Mini (Roche Diagnostics GmbH, Mannheim, Germany), and then centrifuged (20 400 *g* at 4°C for 10 minutes). All specimens underwent sodium dodecyl sulfate-polyacrylamide gel electrophoresis (SDS-PAGE) and were subsequently transferred onto polyvinylidene difluoride membranes. The membranes were initially probed with anti-heme oxygenase 1 antibody (1:1000 dilution, # ab13243; Abcam Ltd, Cambridge, MA, USA). The detection was carried out using secondary antibodies conjugated with horseradish peroxidase. The signal intensity was quantified using LAS 4000 Mini and Multi Gauge software version 3.0 (Fujifilm, Tokyo, Japan), as detailed in a previous report.^14^

### Cell culture and reporter assay

RAW 264.7 cells (TIB-71) were purchased from ATCC and cultured in Dulbecco's Modified Eagle Medium (DMEM) containing 10% heat-inactivated fetal bovine serum (FBS) and 1% (v/v) penicillin/streptomycin. The cells were maintained at 37°C in a 5% CO_2_ atmosphere. To introduce antioxidant response element (ARE)-nanoluciferase (ARE-Nluc) into the cells, the plasmid pCDH ARE-Nluc GFP-T2A-Puro was constructed by linking ARE (JQ858521, Promega) and nanoluciferase (JQ437370, Promega) to the pCDH-CMV7-MCS-EF1-GFP-T2A-Puro (CD513B-1, SBI) backbone, generating a nonreplicative lentivirus. To produce the lentivirus, 293TN cells (LV900A-1, SBI) were transfected with the plasmid pCDH ARE-Nluc GFP-T2A-Puro, along with the packaging plasmid pPACKH1 (LV500A-1, SBI). This lentivirus was used to infect RAW 264.7 cells to introduce ARE-Nluc. The transduced cells were selected with 10 μg/mL puromycin, and green fluorescent protein (GFP) expression was confirmed in all cells after 1 week (RAW-ARE).

For cell viability and reporter assays, RAW-ARE cells were seeded into 96-well white plates with clear bottoms at 1 × 10^4^ cells per well. The 10 mg/mL PS suspended in 0.1% Tween 80 water solution was diluted to 2 mg/mL using DMEM medium and serial 5-fold dilutions were prepared using DMEM medium. The size distribution of each polystyrene particle in the dispersion with DMEM medium was measured by DLS. After 12 hours, each dilution of a maximum concentration of 2 mg/mL and each serial 5-fold dilution was added to the 96-well white plates in an amount equal to the amount of medium in the well (in triplicate wells for both ATP and Nluc measurements). After 24 hours, 50 μL of CellTiter-Glo® 2.0 Cell Viability Assay reagent (Promega) was added to each well to measure ATP activity, and 50 μL of Nano-Glo® Luciferase Assay System reagent (Promega) was added to each well. These activities were immediately measured using a luminometer (Luminescencer JNII RAB-2300; ATTO, Tokyo, Japan). The maximum concentration to which the cells were exposed was 1 mg/mL (0.1 mg per well), which is equivalent to a rat intratracheal instillation dose of 10 mg/rat (assumption: 1 × 10^6^ cells in rat lung); if a human were exposed to 3 mg/m^3^, the deposition would be equivalent to approximately 18 years of exposure, using our previous estimation.^15^

### Histopathology

Formaldehyde-fixed left lung tissue was embedded in paraffin, sectioned at a thickness of 4 μm, and then stained with hematoxylin and eosin (HE) staining.

### Statistical analysis

Statistical analysis was done using IBM® SPSS® software (IBM Corporation, Chicago, IL, USA). *P* values <.05 were considered statistically significant. The Dunnett test was used to detect differences between the exposures (0.2 and 1.0 mg) and negative control group. Tukey honestly significant difference (HSD) tests were appropriately used to detect differences between exposure to polystyrene with different surface functional groups. Differences between 2 groups (negative control and 1.0 mg) were analyzed for statistical significance using the Welch *t* test.

## Results

### Characterization of polystyrene suspension

**Figure 1:**
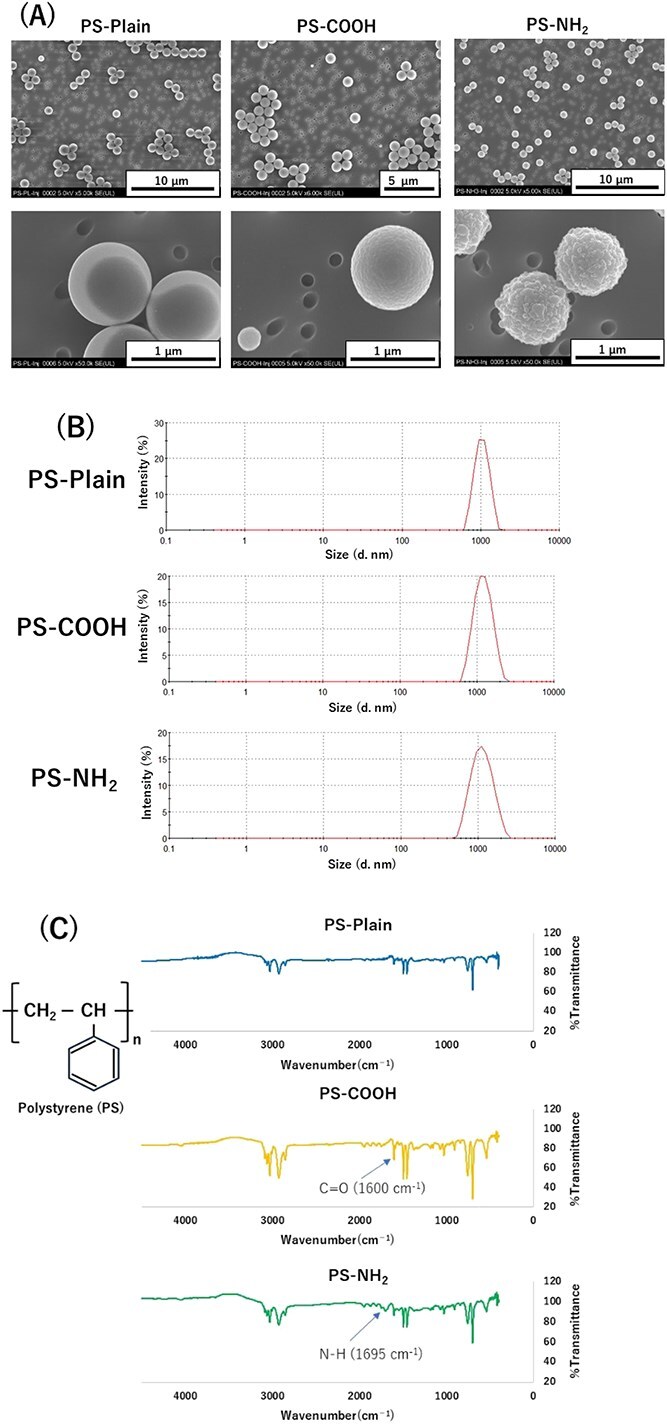
Characterization of polystyrene suspensions. (A) Scanning electron microscopic observation of polystyrenes with different surface functional groups in suspension used in the intratracheal instillation in rats. (B) Particle size distribution of the polystyrenes by dynamic light scattering in suspension used in the intratracheal instillation in rats. (C) Fourier transform infrared (FT-IR) spectra of pristine polystyrene (PS-Plain), amino-functionalized polystyrene (PS-NH_2_), and carboxy-functionalized polystyrene (PS-COOH).

SEM images (Figure 1A) of the suspensions of polystyrene with different functional groups showed good dispersion at the high dose. The surface of PS-Plain was smooth, but that of PS-NH_2_ was rough. There was also a single peak in the particle size distribution of polystyrenes in the suspension, and the agglomerate average diameter was 1.027, 1.172, and 1.071 μm as measured by DLS, respectively (Figure 1B). Even the particle size distribution of polystyrene in DMEM medium with FBS had a main peak around 1 μm, with mean aggregate diameters of 1.505, 1.315, and 1.314 μm, respectively, as measured by DLS (Figure S1). Each polystyrene suspended in 0.1% Tween 80 water solution was analyzed for pH and surface charge. Although the pH was slightly higher for PS-NH_2_, the zeta-potential was around 0 for all the polystyrenes, with little difference observed (Table S1). The FT-IR spectra of PS-Plain, PS-COOH, and PS-NH_2_ are shown in Figure 1C. The transmission at 1600 cm^−1^ for C = O stretching band in the spectrum of PS-COOH and 1695 cm^-1^ for N – H bond in the spectrum of PS-NH_2_ clearly increased compared with that of PS-Plain, respectively. These changes indicated qualitative carboxylation and amination in the presence of the surface particles of PS-COOH and PS-NH_2_, respectively.

### Inflammatory response following intratracheal instillation

**Figure 2:**
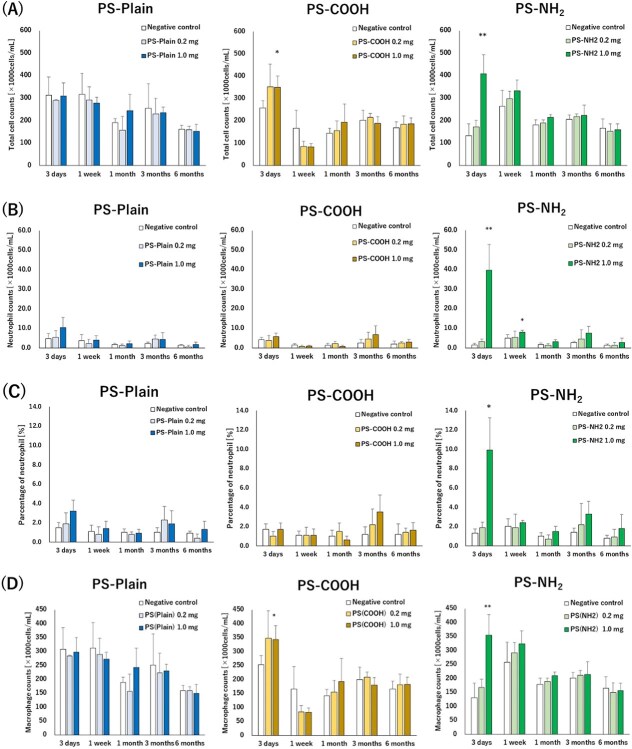
Cell analysis in bronchoalveolar lavage fluid (BALF) following intratracheal instillation of polystyrenes. (A) Total cell counts in BALF. (B) Neutrophil counts in BALF. (C) Percentage of neutrophils in BALF. (D) Macrophage counts in BALF. Intratracheal instillation of amino-functionalized polystyrene (PN-NH_2_) induced a transient influx of inflammatory cells in BALF from 3 d to 1 wk after instillation. Dunnett tests were performed to compare each negative control. Data are presented as mean ± SD for *n* = 5/group (^*^P < .05, ^**^  *P* < .01).

The cell analyses of BALF after exposure to PS-Plain, PS-COOH, and PS-NH_2_ are shown in Figure 2. The total cell and macrophage counts were significantly increased at only 3 days after exposure to PS-NH_2_ and PS-COOH at high doses (Figure 2A,D), the neutrophil counts were increased significantly until 1 week for PS-NH_2_ at high doses, and the neutrophil ratio was significantly increased at 3 days after exposure to PS-NH_2_ at high doses (Figure 2B,C). Dose-dependent changes were observed for both polystyrenes, but the inflammation responses were only in the acute phase.

**Figure 3:**
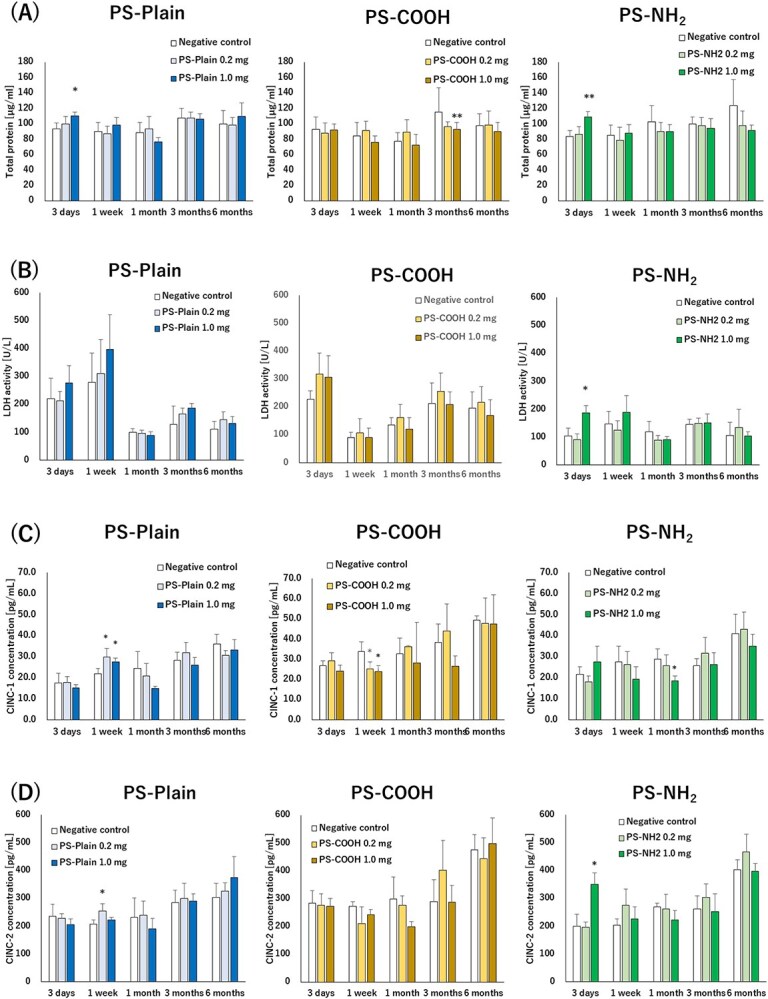
Lung injury markers and cytokine concentration in bronchoalveolar lavage fluid (BALF) following intratracheal instillation of polystyrenes. (A) Total protein concentration in BALF. (B) Lactate dehydrogenase (LDH) activity in BALF. (C) Concentration of CINC-1 in BALF. (D) Concentration of CINC-2 in BALF. Intratracheal instillation of pristine polystyrene (PS-Plain) and amino-functionalized polystyrene (PS-NH_2_) induced transient lung injury in BALF 3 d after instillation. Intratracheal instillation of polystyrenes induced a transient increase of CINC-1 and CINC-2 in BALF during the acute phase. Dunnett tests were performed to compare each negative control. Data are presented as mean ± SD for *n* = 5/group (^*^P < .05, ^**^P < .01). CINC, cytokine-induced neutrophil chemoattractant.

The lung injury marker and cytokines of BALF after exposure to PS-Plain, PS-COOH, and PS-NH_2_ are shown in Figure 3. The total protein concentration in BALF was significantly increased in the high-dose group after 3 days, not only for PS-NH2 but also for PS-Plain (Figure 3A). LDH activity in BALF was significantly increased only in the high-dose group at 3 days after exposure to PS-NH2 compared with the negative control group (Figure 3B), but these lung injury markers were changed only in the acute phase. In BALF, CINC-1 showed a significant increase at both low and high doses of PS-Plain at 1 week after exposure (Figure 3C), whereas CINC-2 showed a significant increase at low doses of PS-Plain at 1 week after exposure and at high doses of PS-NH_2_ at 3 days (Figure 3D). Neither CINC-1 nor CINC-2 showed a significant increase after 1 month. No significant differences in rat body weight were observed following intratracheal instillation (data not shown).

### Histopathological findings

**Figure 4:**
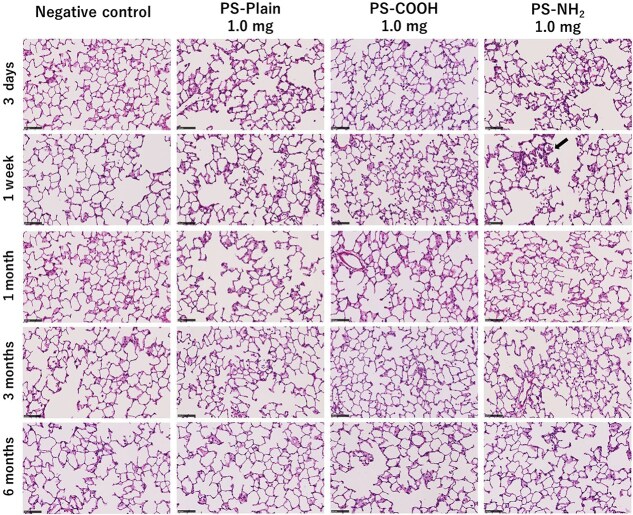
Hematoxylin and eosin staining of lung sections following inhalation exposure of polystyrene at each time course. Mild inflammation, mainly neutrophils and alveolar macrophages, was observed until 1 wk after exposure to amino-functionalized polystyrene (PS-NH_2_; black arrows). No fibrosis was observed in the chronic phase. Scale bar: 100 μm.

Figure 4 shows the histopathological findings in the lung following intratracheal instillation of polystyrenes with different surface functional groups in each high-dose group. There was a very slight influx of inflammatory cells into the alveolar space 3 days after intratracheal instillation. The influx of inflammatory cells with PS-NH_2_ was stronger than with PS-Plain and PS-COOH, and was observed for up to about 1 week, but there was no persistent inflammation or fibrosis in any of the exposure groups in the chronic phase.

### Oxidative stress in lung tissue and cell line following exposure to polystyrene with different functional groups

**Figure 5:**
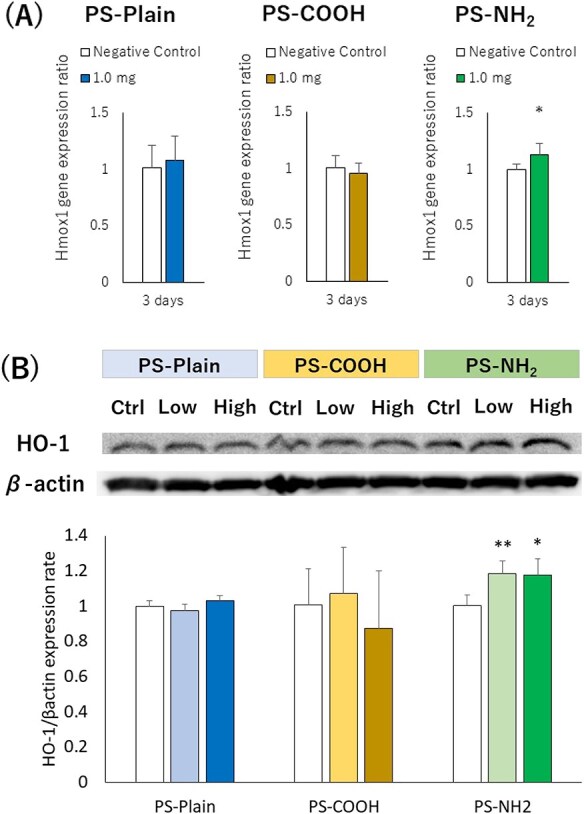
HO-1 expression in the lung tissue at 3 d after the intratracheal instillation of polystyrene with different functional groups. (A) Hmox1 gene expression in qRT-PCR in the lung tissue at 3 d after exposure. (B) HO-1 expression in western blotting in the lung tissue at 3 d after exposure. Only amino-functionalized polystyrene (PS-NH_2_) showed a significant increase in Hmox1 gene and HO-1 expression. Welch *t* tests were performed to compare differences between the 2 groups (negative control and 1.0 mg) in qRT-PCR. Dunnett tests were performed to compare each negative control in western blotting. Data are presented as mean ± SD for *n* = 5/group (^*^P < .05, ^**^P < .01). Hmox1, Heme Oxygenase-1 gene; HO-1, Heme Oxygenase-1; qRT-PCR, quantitative real-time polymerase chain reaction.

**Figure 6:**
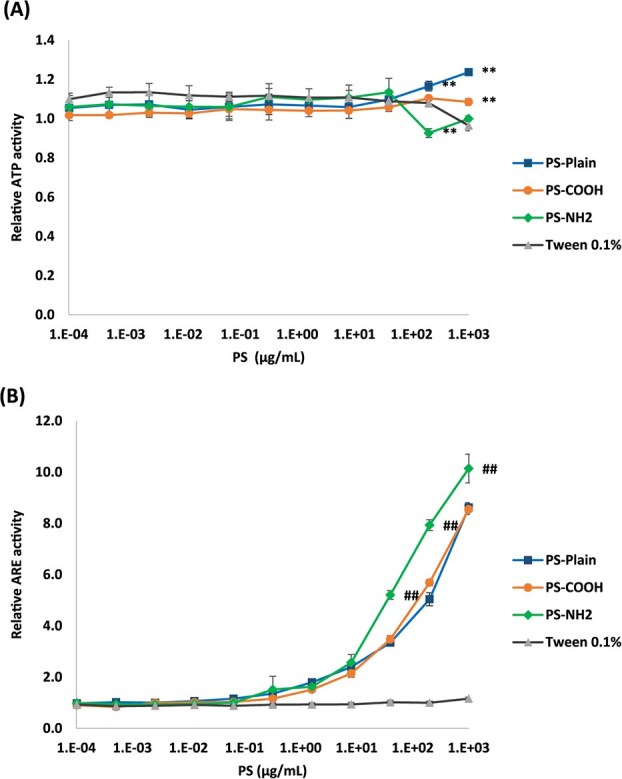
Cell viability and oxidative stress activity in RAW cells. (A) Relative ATP activity in RAW cells at 24 h after treatment with polystyrene having different functional groups. (B) Relative antioxidant response element (ARE) activity in RAW cells at 24 h after treatment with polystyrene having different functional groups. Exposure to amino-functionalized polystyrene (PS-NH_2_) in the RAW cell line significantly decreased ATP compared with 0.1% Tween 80 water solution as a negative control, and increased ARE promoter activity in a dose-dependent manner compared with pristine polystyrene (PS-Plain) and carboxy-functionalized polystyrene (PS-COOH). Tukey honestly significant difference (HSD) tests were performed to compare polystyrene with different functional groups. Data are presented as mean ± SD for *n* = 3/group (vs negative control: ^*^P < .05, ^**^P < .01; vs PS-plain and PS-COOH: #*P* < .05, ##*P* < .01).

Figure 5 shows the HO-1 expression in the lung tissue at 3 days after the intratracheal instillation of polystyrene with different functional groups, analyzed by using qRT-PCR and western blotting. Only PS-NH_2_ showed a significant increase in HO-1 expression at low and high doses in both analyses (Figure 5A,B). On the other hand, a significant decrease in the expression of glutamate-cysteine ligase modifier subunit (Gclm) or sulfiredoxin 1 (Srxn1) in qRT-PCR was observed in PS-NH_2_ (Figure S2). To confirm whether oxidative stress is involved in exposure to polystyrene with different functional groups, we used a reporter assay of the ARE as an indicator of oxidative stress, in the RAW cell line. We observed a significant decrease in ATP activity, as an indicator of cell viability, in RAW cells with the treatment of PS-NH_2_ (Figure 6A). Exposure to PS-NH_2_ in the RAW cell line significantly increased ARE promoter activity in a dose-dependent manner compared with PS-Plain and PS-COOH (Figure 6B).

## Discussion

Polystyrene with different surface functional groups was used in this study. It is known that the surface charge differs depending on the surface functional group. In general, NH_2_ groups have a positive charge, and COOH groups have a negative charge. However, the zeta-potential of the polystyrene used in this study did not show much difference in surface charge. It has been reported that the zeta-potential depends on the pH of the solvent and can also change due to interference from surfactants in the solvent.^16^^,^^17^ Shi et al^9^ reported that there were no differences in zeta-potential due to differences in functional groups in water or culture medium. In contrast, in the FT-IR analysis of the polystyrene powder in the present study, the NH_2_ groups of PS-NH_2_ and the COOH groups of PS-COOH were more C = O and N – H functionalized compared with PS-Plain, respectively. The surface properties were also different in the electron microscope images, so it is inferred that the surface properties of the polystyrene used in this study were different.

A single intratracheal instillation of polystyrene with different functional groups was conducted in this study. The exposure dose for the intratracheal instillation was set at a low dose of 0.2 mg/rat and a high dose of 1.0 mg/rat. These doses were based on the results of previous reports of a single intratracheal instillation, which showed that the minimum dose at which inflammation was observed with highly harmful particles was 0.2 mg,^18^ and that even less harmful particles caused persistent lung inflammation and lung damage at doses above 1.0 mg.^19^^,^^20^ This study was conducted to evaluate the inflammatory and fibrotic potential of chemicals and does not necessarily reproduce human pathological conditions. Inhalation exposure can be evaluated as the reproduction of human pathological conditions, but we conducted intratracheal instillation by adjusting physicochemical properties such as particle size and dosage to approximate human pathological conditions as much as possible.

The present study aimed to evaluate chronic lung disorder following intratracheal instillation of polystyrene with different functional groups. We evaluated the lung disorder based on the results of previous intratracheal instillation of inorganic chemicals. Under the mentioned exposure conditions, inhaled chemicals with high lung toxicity (chronic effects), that is, those that cause fibrosis or tumor formation in the long term, caused inflammation and fibrosis that persisted for more than 3 months, whereas recovery within 1 month was seen for the chemicals with low lung toxicity. Therefore, the borderline for evaluation of lung disorder is the presence or absence of persistent inflammation for 1 to 3 months after exposure.^15^^,^^21^^-^^23^

It has been reported that chemicals with high toxicity, such as asbestos and crystalline silica, have persistent inflammatory responses for more than 1 month after intratracheal instillation.^24^^-^^26^ In the present study of intratracheal instillation of polystyrenes with different surface functional groups in rats, the number of neutrophils in the BALF was significantly increased for up to 1 week after instillation with PS-NH_2_, but only transiently increased at 3 days after exposure to PS-Plain and PS-COOH. In the observation of lung pathological specimens, the influx of inflammatory cells into the alveolar space was observed for up to 1 week after exposure to PS-NH_2_, but there was no inflammatory cell infiltration in the alveolar interstitium. The lung inflammation was transient with all types of polystyrenes, and no fibrosis was observed at 3 to 6 months after exposure. In another report, intratracheal instillation of similarly NH_2_-modified polystyrene in rats showed lung inflammation in the acute phase 24 hours after exposure,^11^ and multiple intratracheal instillations of polystyrene or polypropylene have been shown to increase neutrophil counts and inflammatory cytokines from 14 to 21 days after the initial exposure.^10^^,^^27^ Considering that all the types of polystyrenes used in the present study showed transient inflammation, if microplastics and inorganic substances have the same mechanism of lung injury, the risk of lung disorder in the chronic phase caused by the different functional groups of polystyrene may be low.

On the other hand, inflammatory changes due to differences in functional groups were observed during the acute phase. Analysis of CINC, which is known to function as a neutrophil migration and activation factor, showed that CINC-1 (CXCL1) and CINC-2 (CXCL3) had tendencies to increase at 3 days after the instillation of PS-NH_2_. There was a significant increase in CINCs at 1 week after exposure to PS-Plain. However, there was no significant increase in neutrophils in the BALF at 1 week after exposure to PS-Plain, and there was also no dose-dependent change. It is thought that the increases in CINC were not significant for inflammatory changes due to exposure to PS-Plain at 1 week. There was also a significant increase in LDH activity and total protein concentration, which are indicators of lung injury, 3 days after the instillation of PS-NH_2_, whereas PS-Plain showed a significant increase in total protein concentration only, and PS-COOH showed no lung injury, indicating that PS-NH_2_ tended to cause the strongest injury during the acute phase.

Other reports have also found differences in response due to differences in functional groups during the acute phase. Kim et al^11^ reported that in an intratracheal instillation in rats, PS-NH_2_ increased interleukin-1b and CINC-3 in BALF, whereas PS-COOH did not show a significant increase in them. In the cell culture of this study, PS-NH_2_ caused a significant decrease in ATP activity, which correlated with cell viability, in macrophage RAW cells. Other reports have also shown that polystyrene with the NH_2_ functional group is highly cytotoxic,^28^^,^^29^ and it was reported that PS-NH_2_ was more cytotoxic than PS-Plain or PS-COOH. It has been reported that the mechanism of toxicity with different functional groups is due to the difference in charge of the polystyrene particles, which affects adhesion to and uptake by cells. Chen et al^30^ reported that PS-NH_2_ was the polystyrene most taken up by RAW cells, and damage to the cell membrane was observed more than with unmodified PS or PS-COOH, suggesting that the positive charge of PS-NH_2_ affects the cytotoxicity. However, these differences in functional groups in terms of acute inflammation are not pathological precursors of chronic effects, so for human exposure at the expected level, even if there are harmful effects, they are likely to be minor differences.

We focused on oxidative stress in acute lung inflammation and lung injury caused by differences in functional groups of polystyrene. Real-time PCR was used to measure HO-1, Gclm, and Srxn1 in lung tissue, and only HO-1 was significantly increased in PS-NH_2_ compared with the control group. HO-1 expression in western blotting was also elevated only for PS-NH_2_ in rat lung tissue 3 days after intratracheal instillation. The tendency for increased oxidative stress was also observed in RAW cells, and the expression of ARE, an oxidative stress marker, was most significantly increased in PS-NH_2_. ARE is considered an indicator of oxidative stress because it induces the antioxidants HO-1, Gclm, and Srxn1.^31^^-^^33^ In this study, significant gene expression was observed only for HO-1, and significant expression was also observed at the protein level, suggesting that PS-NH_2_ induced oxidative stress in the lung during the acute phase. It has also been reported that microplastics cause oxidative stress.^27^^,^^29^^,^^34^ Jeon et al^29^ reported that only polystyrene with NH_2_ groups, compared with polystyrene without modification and with a COOH group, increased reactive oxygen species (ROS) in BEAS-2B cells. Guo et al^35^ reported that HO-1 was produced via activation of the nuclear factor erythroid 2-related factor 2 (Nrf2) pathway due to ROS production in HepG2 cells exposed to polystyrene nanoparticles. They also reported that mitochondrial damage, the release of inflammatory cytokines, and ROS production due to exposure to polystyrene nanoparticles were alleviated by treatment with *N*-acetyl-l-cysteine. Considering that these reports indicate a relationship between oxidative stress and inflammation due to exposure to polystyrene, the difference in oxidative stress potential according to the surface functional groups of polystyrene may have influenced the difference in acute lung inflammation potential in the present study.

Polystyrene with different surface functional groups was intratracheally instilled into rats in this study. Various environmental pollutants other than microplastics exist in the general atmosphere, not just in the workplace.^36^ Since changes in the surface functional groups of microplastics affect the adhesiveness of chemicals,^37^^,^^38^ the toxicity of microplastics may increase due to interactions with chemicals suspended in the atmosphere. It has been reported that the adhesiveness of chemicals to microplastics enhances allergic reactions.^39^ In assessing the health effects of airborne microplastics, it is necessary to clarify the additive and synergistic effects of air pollutants in the future.

In this study, we conducted intratracheal instillation in rats using polystyrene with different surface functional groups, and evaluated lung disorders in rats during the acute and chronic phases. Our results showed that, in the acute phase, PS-NH_2_ had the highest oxidative stress and caused lung inflammation compared with PS-Plain and PS-COOH. However, the polystyrenes caused only transient lung inflammation, and no fibrosis progression was observed in the chronic phase. These results suggest that lung disorders caused by differences in the surface functional groups of polystyrene may not be severe.

## Supplementary Material

Web_Material_uiaf006

## Data Availability

The datasets created and/or analyzed during the current study are available from the corresponding author on reasonable request.
