# Supplementary material for: Comparison of lung disorders following intratracheal instillation of polystyrene microplastics with different surface functional groups
Source: J Occup Health. 2025 Feb 3;67(1):uiaf006. doi: 10.1093/joccuh/uiaf006 (PMC11894927; doi:10.1093/joccuh/uiaf006)

Supporting Information

Supplementary Table 1　Zeta potential and pH of polystyrene

|  | PS-Plain | PS-COOH | PS-NH_2_ |
| --- | --- | --- | --- |
| ζ-potential (mV) | -0.44±0.08 | -0.01±2.80 | 0.50±1.17 |
| pH | 6.74 | 6.64 | 7.44 |

Supplementary Figure 1.

Particle size distribution of the polystyrenes by dynamic light scattering in DMEM with FBS including 0.1% Tween 80 water solution used in the cell culture. DMEM, Dulbecco's Modified Eagle Medium.


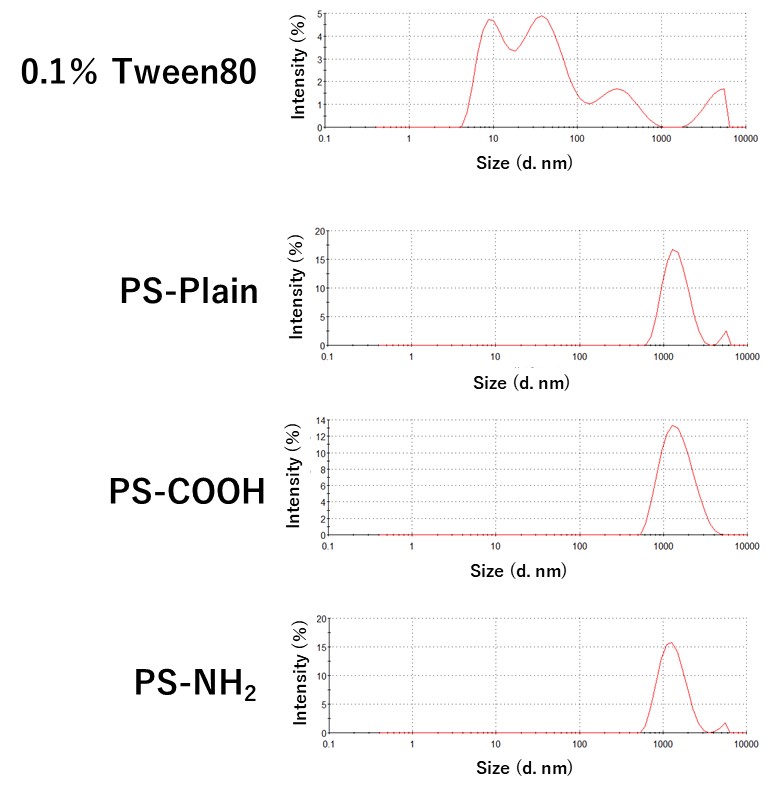


Supplementary Figure 2

Oxidative stress-related gene expressions in qRT-PCR in the lung tissue at 3 days after the intratracheal instillation of polystyrene with different functional groups. (A) Glutamate-Cysteine Ligase Modifier Subunit (*Gclm*) gene expression. (B) Sulfiredoxin 1 (*Srxn1*) gene expression. In each polystyrene-exposed group, no significant increase in *Gclm* or *Srxn1* was observed compared with each control group, conversely, *Gclm* and *Srxn1* gene expressions were decreased in PS-Plain and PS-NH_2_ exposure groups. Welch's t-tests were performed Differences between two groups (negative control and 1.0 mg) in qRT-PCR. Data are presented as mean ±SD for n= 5/group (* p < 0.05, ** p < 0.01). qRT-PCR, quantitative real-time polymerase chain reaction.


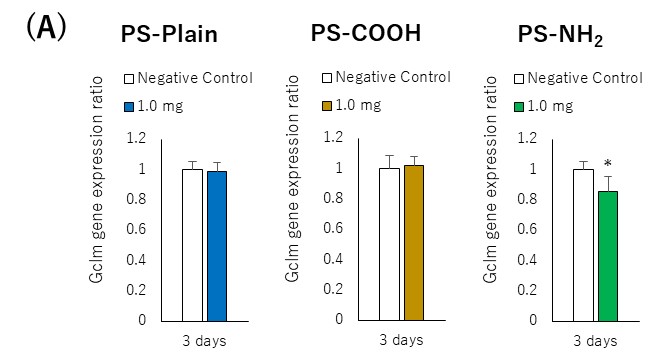


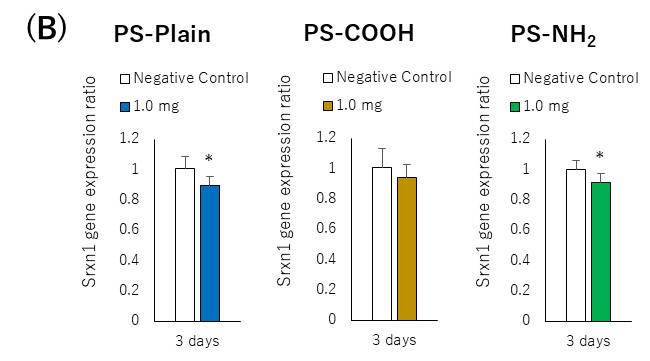

Supplement: Web_Material_uiaf006 [file web_material_uiaf006.zip › Supplementary file revised.v2.docx]
